# The Blurred Future of Adolescent Gamblers: Impulsivity, Time Horizon, and Emotional Distress

**DOI:** 10.3389/fpsyg.2017.00486

**Published:** 2017-04-03

**Authors:** Giovanna Nigro, Marina Cosenza, Maria Ciccarelli

**Affiliations:** Department of Psychology, Università degli Studi della Campania Luigi VanvitelliCaserta, Italy

**Keywords:** gambling, adolescence, impulsivity, delay discounting, temporal perspective, depression, anxiety, negative affect

## Abstract

The main purpose of this study was to investigate the interplay of functional and dysfunctional impulsivity, delay discounting, time perspective, and emotional negative states on gambling severity in Italian adolescents. A second aim of the study was to analyze the developmental trajectories of gambling involvement, functional and dysfunctional impulsivity, delay discounting, consideration of future consequences, and negative affectivity in a cross-sectional perspective. One thousand and ten Italian adolescents aging between 12 and 19 years were administered the South Oaks Gambling Screen Revised for Adolescents (SOGS-RA), the Functional and Dysfunctional Impulsivity Scale (FDIS), the Monetary Choice Questionnaire (MCQ), the Consideration of Future Consequences Scale (CFC-14), and the Depression, Anxiety and Stress Scales-21 (DASS-21). Data analyses were conducted using correlational analysis, Chi-square test, analysis of variance, and hierarchical regression analysis. Results indicated that, relative to non-gamblers and non-problem gamblers, at-risk and problem gamblers showed higher levels of impulsivity, steeper delay discounting, shorter time horizon, and reported experiencing significantly higher levels of depression, anxiety, and stress. Results of hierarchical regression analysis, with SOGS-RA scores as the dependent variable, and gender, age, FDIS, MCQ, CFC-14, and DASS-21 scores as independent variables, indicated that, along with gender and age, low scores of future orientation and high scores of dysfunctional impulsivity, depression, anxiety, present orientation, and delay discounting significantly predicted gambling severity. These findings provide further evidence that the higher the gambling involvement, the greater the tendency to devalue delayed rewards and to focus on the immediate consequences of one's behavior. Interestingly, for the first time these results reveal an association between gambling severity and both dysfunctional impulsivity and negative affective states across adolescence. Finally, results of cross-sectional analyses suggest that gambling severity contributes more than age in shaping the developmental trajectories of functional and dysfunctional impulsivity, delay discounting, time perspective, and negative affective states.

## Introduction

In the last decades gambling addiction has become a serious public health issue. Mainly due to the increasing availability of online gambling and the similarity between modern forms of gambling and other familiar technology-based games, the prevalence of disordered gambling will predictably increase further in the near future (Donati et al., 2013; McCormack et al., 2014; Delfabbro et al., 2016). Recently, Gainsbury et al. (2016b) have demonstrated that for a large proportion of at-risk and problem gamblers the exposure and the engagement with social media advertisements for gambling worsened their problems (see also Gainsbury et al., 2016a).

In this backdrop, adolescent participation in gambling activities is of particular concern, given that some risk factors for disordered gambling are so manifest during adolescence, that adolescence by itself may be regarded as a risk factor for the onset and the development of problematic gambling (Messerlian et al., 2004, 2005; van den Bos et al., 2013).

Indeed, large-scale international prevalence surveys and meta-analytic studies have shown that 10–15% of adolescents are at risk for developing gambling problems and 3–8% can be considered having serious gambling problems (see Blinn-Pike et al., 2010; Ladouceur et al., 2013). A recent review on the prevalence of adolescent problem gambling across five continents reported that 0.2–12.3% of youth meet diagnostic criteria for problem gambling (Calado et al., 2016). In spite of gambling is an illegal activity in Italy under the age of 18, some studies on Italian adolescents have found that 16–17% of high school students were at-risk of developing problem gambling, and 7–8% problem gamblers (Chiesi et al., 2013; Cosenza et al., 2014; Cosenza and Nigro, 2015).

Even if studies on adolescent gambling increased in the last years, research on risk factors from early to late adolescent gambling remains very scarce. This is even more surprising since several studies have highlighted that adolescents represent a high vulnerable population and research has demonstrated that, other things being equal, severe gambling-related difficulties in adulthood steam from early gambling problems (Blinn-Pike et al., 2010; Volberg et al., 2010; Olason et al., 2011; Cosenza et al., 2014; Gupta and Derevensky, 2014).

While there are several potential factors leading to the onset and development of problematic gambling, the research on the identification of risk factors associated with youth disordered gambling is still limited in quantity (Shead et al., 2010; Scholes-Balog et al., 2014). Furthermore, to date the interplay of different risk factors on adolescent problem gambling has not been adequately taken into account (Cosenza and Nigro, 2015).

Although the etiology of gambling disorder is complex and multifaceted, several studies have identified impulsivity as the most robust characteristic associated with disordered gambling (MacKillop et al., 2014). Interestingly, prospective investigations have indicated that high impulsivity during early adolescence predicts later gambling problems (Pagani et al., 2010; Shenassa et al., 2012; Slutske et al., 2012).

Impulsivity describes a constellation of heterogeneous traits or behavioral dispositions that includes inability to take into account the future consequences of current behavior and the tendency to devalue delayed rewards. Acting without considering future consequences has been considered one of the potential determinants of impulsive behavior (Whiteside et al., 2005; see also Sharma et al., 2013). Likewise, delay discounting, that is the relative preference for small immediate rewards, has been considered a behavioral index of impulsivity (Ainslie, 1975; Madden et al., 2009; see also Amlung and MacKillop, 2011; Gray and MacKillop, 2014). Studies examining the relation between gambling and delay discounting among late adolescents indicated that, relative to non-problem gamblers, young problem gamblers more rapidly discounted delayed monetary outcomes (for exception see Holt et al., 2003; MacKillop et al., 2006; Cosenza and Nigro, 2015; Nigro and Cosenza, 2016).

The association between pathological gambling and shortened time horizon was first investigated by Hodgins and Engel (2002). Subsequent studies further supported the existence of a positive association between disordered gambling and insensitivity to future consequences among both adult (Toplak et al., 2007; MacLaren et al., 2012; Ciccarelli et al., 2016b) and adolescent gamblers (however, for different results, see MacKillop et al., 2006; Cosenza and Nigro, 2015; Cosenza et al., 2016).

Finally, as indicated by earlier studies, negative emotional states, such as depression, anxiety, and stress, are significant correlates of problematic gambling (Blaszczynski and McConaghy, 1989; Coman et al., 1997; Blaszczynski and Nower, 2002; El-Guebaly et al., 2006; Kim et al., 2006; Ladouceur et al., 2006; Johansson et al., 2009; Barrault and Varescon, 2013; Lorains et al., 2014; Dowling et al., 2015; Raylu et al., 2016; Toneatto and Pillai, 2016). In particular, some epidemiological studies indicated that problematic gambling is often associated with mood disorders (Griffiths, 1995; see also Lorains et al., 2011), as well as that pathological gamblers in treatment frequently suffer from clinical depression (i. e., Ladouceur et al., 2006). Nower and Blaszczynski (2010) hypothesized that gambling contributes to alleviate negative emotional states or boredom (Wulfert et al., 2005; Wood and Griffiths, 2007; see also Stewart et al., 2008), whereas Gee et al. (2005) observed that gambling increases anxiety.

From the few studies investigating the co-occurrence of negative affects and gambling in adolescence emerged that, relative to both non-gamblers and social gamblers, adolescent problem gamblers have higher rates of depression, females have significantly higher rates of depression than males, and older adolescents score higher than younger (Nower et al., 2004). Furthermore, compared to non-gamblers, social and at-risk gamblers, adolescent problematic gamblers report higher level of both state and trait anxiety and social stress, with females obtaining higher scores than males (Ste-Marie et al., 2006). In a sample of young online gamblers Matthews et al. (2009) found that problem gambling was significantly predicted not only by negative mood states after gambling, but also by negative mood states in general. More recently, in a longitudinal study involving adolescents and early adults, Dussault et al. (2011) demonstrated that the association between depression and problematic gambling in adolescence steams mainly from impulsivity. In addition, the mechanisms explaining the association between the two disorders vary as a function of developmental stages.

Although evidences from previous research support the idea that there could be a complex interplay among problematic gambling, impulsivity, “myopia for the future,” and negative emotional states in adolescence, to date no study has ever examined the interrelationship among these variables all together.

The main aim of the present study was to investigate the interplay among impulsivity, delay discounting, time perspective, and negative affectivity in a large sample of adolescents aging between 12 and 19 years. A second aim of the present study was to analyze the developmental trajectories of gambling involvement, functional and dysfunctional impulsivity, delay discounting, consideration of future consequences, and negative affectivity in a cross-sectional perspective.

In line with previous research on both adults and adolescents, it was expected that female adolescents would be less likely to report gambling-related problems than male adolescents. Moreover, it was hypothesized that the more severe the gambling involvement is, the higher the level of impulsivity, the steeper the delay discounting rates, and the shorter the time horizon are. Finally, it was also hypothesized that, relative to other groups, at-risk and problem gamblers would show more severe depression, anxiety, and stress symptoms.

## Methods

### Participants

One thousand and ten Italian students (47,5% males) aged between 12 and 19 years (Mean age = 15.37 years; *SD* = 2.05) attending public middle (14.2%) or high school (58.4% lyceum and 27.4% technical and trade school) in Southern Italy took part in the study. They were administered the South Oaks Gambling Screen Revised for Adolescents (Winters et al., 1993, 1995; Italian version: Colasante et al., 2013; SOGS-RA), the Functional and Dysfunctional Impulsivity Scale (FDIS; Dickman, 1990), the Monetary Choice Questionnaire (Kirby and Marakovic, 1996; Kirby et al., 1999; MCQ), the Consideration of Future Consequences Scale (Joireman et al., 2012; Italian validation: Nigro et al., 2016; CFC-14), and the Depression, Anxiety and Stress Scales-21 (Lovibond and Lovibond, 1995; Italian validation: Bottesi et al., 2015; DASS-21). Participants did not receive anything for participating in the study. The authors administered the questionnaires. For each measure participants received detailed written instructions. Participants were allowed to ask any questions about the questionnaires, if any.

### Measures

Adolescent gambling behavior was measured through the SOGS-RA, the most widespread self- report instrument for assessing the prevalence of problem gambling in adolescence. The questionnaire is made up of 12 scored items measuring gambling behavior and gambling-related problems during the past 12 months. The total score ranges from a minimum of 0 to a maximum of 12. The un-scored SOGS-RA items request participants to indicate, among others, the frequency of participation in different gambling activities, the largest amount of money gambled in 1 day, and parental involvement in gambling. In addition, we asked participants to specify the primary motives for gambling from a list (Volberg, 1993). The Italian version of the SOGS-RA was found to have acceptable internal reliability (α = 0.78; Colasante et al., 2013).

The FDIS is a 23 items self-report questionnaire assessing functional and dysfunctional impulsivity. The Functional Impulsivity scale (FI) consists of 11 items measuring the tendency to act quickly without planning when the situation demands it for personal gain. The Dysfunctional Impulsivity scale (DI) consists of 12 items assessing the tendency to engage in rapid, error-prone information processing in situations where slower methodical approaches are required. Respondents are asked to indicate the extent to which they agree with each statement on a 5-point scale ranging from 1 (*strongly disagree*) to 5 (*strongly agree*). In the present study, Cronbach's alpha for the functional and dysfunctional scales was 0.71 and 0.76, respectively.

The MCQ is a measure of delayed reward discounting that presents participants with 27 hypothetical choices between a smaller reward available immediately, and a larger reward available at some point in the future, with delays ranging from 7 to 186 days. The 27 items are grouped into three categories on the basis of the approximate magnitudes of the delayed rewards. The three levels of magnitude are: small ($25–$35), medium ($50–$60), and large ($75–$85). Participants are instructed to respond in the same manner as they would with real money. The pattern of responding can be used to determine an estimate of the participant's overall discounting rate parameter (*k*), as well as temporal discounting of rewards at the three different levels of magnitude (*k* small, *k* medium, and *k* large). The higher the *k*-values, the greater the proportion of choices for the smaller immediate monetary rewards. Calculating separate discount rates for each level of magnitude allows estimating the magnitude effect on discount rates, i. e., the tendency for discount rates to decrease as a function of reward level (Green et al., 1981).

The CFC-14 is a 14-item scale that was developed to measure individual differences in the extent to which people evaluate the immediate as opposed to distant implications of current behaviors and events. Responses are made with a 7-point Likert scale ranging from 1 (*extremely uncharacteristic of me*) to 7 (*extremely characteristic of me*). The CFC-14 is a two factors scale with two dimensions, one assessing consideration of immediate consequences (CFC-I), the other tapping consideration of future consequences (CFC-F). The Cronbach's alphas for the Immediate and Future scales were 0.84 and 0.83, respectively, in a large sample of Italian adolescents (Nigro et al., 2016).

The DASS-21 is a self-report measure assessing three related negative affective states, namely depression, anxiety, and stress. The Depression scale comprises items that assess symptoms characteristically associated with dysphoric mood, such as sadness, worthlessness, lack of interest or involvement, and low self-esteem. The Anxiety scale taps signs of physical arousal, symptoms of panic attacks, as well as subjective experience of fear. The Stress scale assesses symptoms, such as difficulty relaxing, impatience, and being easily upset, irritable, or overreactive. Respondents are asked to indicate how much each statement applied to them during the previous week on a 4-point Likert scale ranging from 0 to 3. Higher scores indicate severe emotional distress. Cronbach's alphas were, respectively, 0.82 for the depression subscale, 0.74 for the anxiety dimension, 0.85 for the stress subscale, and 0.90 for the full scale (Bottesi et al., 2015).

### Procedure

The study was approved by the Ethics Committee of the Department of Psychology of the Second University of Naples. Prior to participation, all participants gave written informed consent. For minors, informed consent was obtained from parents. Participants were tested in groups of 10 to 20 at a time in a quiet room in school. Administration of all instruments required from 20 to 30 min.

### Statistical analyses

Data were analyzed with the IBM Statistical Package for the Social Sciences, version 20.0. The alpha significance level was set at *p* < 0.05. All variables were initially screened for missing data, distribution abnormalities, and outliers (Tabachnick and Fidell, 2013). Minor missing data (<2%) for all variables were replaced with means. Responses from the MCQ were analyzed using the approach described by Kirby et al. (1999). Because the *k*-values were positively skewed, a natural log transformation was conducted and used for all analyses. Furthermore, given that the distribution of the SOGS-RA was positively skewed, square root transformation was performed on this variable so that assumptions of normality, linearity and homoscedasticity had been adequately met.

Pearson correlation co-efficients and partial correlations were calculated to examine the relationships among the study variables. For categorical data differences in percentages were compared with the Chi-square test. Univariate and mixed-model ANOVAs were used to assess mean differences on continuous variables. *Post hoc* single comparisons were performed using two-tailed *t*-tests for dependent groups with Bonferroni correction for multiple comparisons (*p* < 0.05). The magnitude effect on the discounting task was examined using paired samples *t*-test. Finally, to reveal potential predictors of gambling behavior and gambling-related problems, we performed a hierarchical regression analysis with SOGS-RA scores as the dependent variable, and gender, age, FDIS, MCQ, CFC-14, and DASS-21 scores as independent variables. In order to control for the presence of multicollinearity, before interpreting the regression coefficients, we calculated the variance inflation factors (VIF), which were below the recommended cutoff of 10 (max. VIF = 1.876; Ryan, 1997).

## Results

The associations among variables were assessed first using Pearson's correlation coefficients. Subsequently, we tested for gender differences through univariate analyses of variance (ANOVAs). Results showed significant gender differences on the SOGS-RA, the FDIS Functional Impulsivity dimension, the three discounting rates of the MCQ, the CFC-14 Immediate subscale, with males outperforming females, and on the three dimensions of the DASS-21, with females scoring higher than males. Since age was positively correlated with SOGS-RA, MCQ, and DASS-21 scores, to ascertain whether the measures correlated even after controlling for gender and age, partial correlations among the measures were calculated (see Table 1).

**Table 1:** **Partial correlations among all variables controlling for gender and age**.

	**2**	**3**	**4**	**5**	**6**	**7**	**8**	**9**	**10**	**11**
1. SOGS-RA	0.079^*^	0.341^**^	0.111^**^	0.108^**^	0.136^**^	0.243^**^	−0.207^**^	0.279^**^	0.273^**^	0.271^**^
**FDIS**										
2. Functional Impulsivity		0.279^**^	0.038	−0.012	0.012	0.157^**^	−0.056	−0.047	−0.038	0.037
3. Dysfunctional Impulsivity		–	0.030	0.054	0.059	0.366^**^	−0.224^**^	0.253^**^	0.229^**^	0.275^**^
**MCQ**										
4. *k* small			–	0.592^**^	0.526^**^	0.110^**^	−0.062	−0.014	0.031	0.047
5. *k* medium				–	0.645^**^	0.074^*^	−0.057	0.020	0.069^*^	0.059
6. *k* large					–	0.069^*^	−0.097^**^	0.049	0.077^*^	0.064^*^
**CFC-14**										
7. Immediate						–	0.031	0.185^**^	0.186^**^	0.203^**^
8. Future							–	0.019	0.013	0.039
**DASS-21**										
9. Depression								–	0.666^**^	0.713^**^
10. Anxiety									–	0.673^**^
11. Stress										–

* p < 0.05;

** *p < 0.01*.

As Table 1 shows, correlations between SOGS-RA, FDIS, MCQ, CFC-14, and DASS-21 scores were moderate to strong in strength.

In accordance with Winters et al.'s original SOGS-RA scoring system (1993, 1995), respondents were classified in the following four categories: *non-gamblers*, that includes individuals who reported no past year gambling, *non-problem gamblers* (score of 0–1), *at-risk gamblers* (score between 2 and 3), and *problem gamblers* (score of 4 or more). Of the total sample, 21.6% were screened as non-gamblers, 51.5% as non-problem gamblers, 19% as at-risk gamblers, and 7.9% as problem gamblers. The percentages of common gambling activities as a function of the relative frequency of participation in each activity during the last twelve months are reported in Table 2. As regards the amount of money invested in a single episode of play results indicated that 15.3% of at-risk and problem gamblers spent 1 Euro or less, 62.6% between 1 and 10 Euros, 15.7% between 10 and 50 Euros, 3.8% between 50 and 100 Euros, and 2.7% more than 100 Euros.

**Table 2:** **Percentages of common gambling activities as a function of frequency (12-months-prevalence)**.

	**Never**	**Less than monthly**	**Monthly**	**Weekly**	**Daily**
Cards	27.05	63.99	20.14	10.52	5.35
Horse or dog races	89.60	4.16	2.34	2.73	1.17
Sports betting	43.56	17.95	9.23	23.28	5.98
Dice	91.42	7.02	1.30	0.13	0.13
Casino	94.80	4.03	1.04	0.13	0.00
Scratch cards	51.63	33.03	9.62	5.07	0.65
Lotteries	76.98	17.04	4.68	1.04	0.26
Bingo	84.79	11.44	2.99	0.65	0.13
Slot machines	89.08	6.76	2.86	0.65	0.65
Skill games	71.91	15.60	5.59	4.55	2.34

In order to determine whether gambling activities varied as a function of gender and age, after collapsing gambling activities in three main categories, namely “offline games only” (74% of participants), “online games only” (1% of participants), “both offline and online games” (25% of participants), data were submitted to Chi-square analyses. Non-gamblers and 23 participants who did not specify the gambling activities in which they engaged were excluded from analyses. Chi-square test revealed no significant differences due to gender (χ^2^ (2, *N* = 769) = 5.87; *p* = .053), nor to age (χ^2^ (14, *N* = 769) = 10.51; *p* = 0.724).

Chi-square test was also used to ascertain whether there was an association between severity of gambling involvement and each motive for gambling. Obviously, participants who reported no past year gambling (non-gamblers) were excluded from analysis. Results indicated that at-risk and problem gamblers gamble significantly more to win money (χ^2^ (2, *N* = 792) = 27.99; *p* < 0.001), for excitement or as a challenge (χ^2^ (2, *N* = 792) = 17.39; *p* < 0.001), to socialize (χ^2^ (2, *N* = 792) = 13.64; *p* < 0.01), and for fun or entertainment (χ^2^ (2, *N* = 792) = 8.45; *p* < 0.05).

Group differences on the FDIS, the MCQ, the CFC-14 scales, and on the DASS-21 scores were tested using mixed model ANOVAs. Gender and age were included as covariates in the analyses. Results of 4 × 2 repeated measures ANOVA, with SOGS-RA group as a between-subjects factor and scores on the two FDIS scales, yielded a significant main effect of SOGS-RA group [*F*_(3, 1004)_ = 32.12; *p* < 0.001; $\eta_{\text{p}}^{2}$ = 0.088]. Furthermore, within-subjects contrasts revealed significant interaction effects between FDIS dimensions and gender [*F*_(1, 1004)_ = 55.20; *p* < 0.001; $\eta_{\text{p}}^{2}$ = 0.052], age [*F*_(1, 1004)_ = 6.67; *p* < 0.01; $\eta_{\text{p}}^{2}$ = 0.007], and SOGS-RA classification [*F*_(3, 1004)_ = 22.19; *p* < 0.001; $\eta_{\text{p}}^{2}$ = 0.062). Over and above gender and age effects, these results indicated that, in general, at-risk and problem gamblers were more impulsive than non-gamblers and non-problem gamblers. Of interest, non-gamblers and non-problem gamblers scored significantly higher on the functional scale than on the dysfunctional one, whereas at-risk and problem gamblers scored significantly lower on the functional impulsivity scale than on the dysfunctional impulsivity dimension.

As regards delay discounting performance, all participants showed higher *k*-values for smaller, compared to larger delayed rewards. All pair-wise differences in *k* between reward magnitudes were highly reliable overall and within the four groups (all *ps* < 0.001).

Choice behavior was analyzed using a 4 × 3 mixed-model ANOVA of group by magnitude (small, medium, and large). The analysis yielded significant main effects due to gender [*F*_(1, 1004)_ = 9.38; *p* < 0.01; $\eta_{\text{p}}^{2}$ = 0.009], age [*F*_(1, 1004)_ = 18.42; *p* < 0.001; $\eta_{\text{p}}^{2}$ = .018], and group [*F*_(3, 1004)_ = 5.32; *p* < 0.01; $\eta_{\text{p}}^{2}$ = 0.016], indicating that males scored higher than females on the MCQ, delay discounting become steeper as a function of age, and at-risk and problem gamblers showed higher rates of delay discounting than did non-gamblers and non-problem gamblers.

Regarding CFC-14 scores, results of a 4 × 2 repeated measures ANOVA yielded a significant main effect of gender (*F*_(1, 1004)_ = 7.69; *p* < 0.01; $\eta_{\text{p}}^{2}$ = 0.008], with males reporting higher scores on the Immediate subscale than females, as well as an interaction effect between SOGS-RA group and the two dimensions of the CFC-14 [*F*_(3, 1004)_ = 30.27; *p* < 0.001; $\eta_{\text{p}}^{2}$ = 0.083], indicating that Immediate scores increase as a function of gambling severity, whereas Future scores decrease according to gambling involvement.

A 4 × 3 repeated measures ANOVA was also conducted on DASS-21 scores. Results indicated significant main effects due to gender [*F*_(1, 1004)_ = 41.04; *p* < 0.001; $\eta_{\text{p}}^{2}$ = 0.039] and SOGS-RA group [*F*_(3, 1004)_ = 31.39; *p* < 0.001; $\eta_{\text{p}}^{2}$ = 0.086], showing that females scored significantly higher than males on the three DASS-21 dimensions, and that negative emotional states increase as a function of gambling severity.

Means and standard deviations by SOGS-RA group are presented in Table 3. To facilitate interpretation, descriptive statistics are reported for the untransformed variables.

**Table 3:** **Means and standard deviations by SOGS-RA groups**.

	**Non-gamblers**		**Non-problem gamblers**		**At-risk gamblers**		**Problem gamblers**		**Total sample**	
	**Mean**	***SD***	**Mean**	***SD***	**Mean**	***SD***	**Mean**	***SD***	**Mean**	***SD***
**FDIS**										
Functional Impulsivity	30.15	6.07	31.52	5.94	32.46	6.84	33.69	6.43	31.57	6.25
Dysfunctional Impulsivity	28.68	6.75	30.12	6.87	34.10	7.01	36.71	7.58	31.09	7.34
**MCQ**										
*k* Total score (overall *k*)	0.02	0.01	0.02	0.02	0.03	0.03	0.04	0.04	0.02	0.02
*k* Small	0.03	0.04	0.04	0.05	0.05	0.06	0.06	0.07	0.04	0.05
*k* Medium	0.02	0.03	0.03	0.03	0.03	0.04	0.05	0.06	0.03	0.03
*k* Large	0.01	0.01	0.02	0.02	0.02	0.04	0.03	0.04	0.02	0.03
**CFC-14**										
Total score	4.88	0.58	4.76	0.66	4.46	0.78	4.11	0.90	4.68	0.72
Immediate	19.86	6.30	20.41	6.39	22.96	7.54	25.13	8.52	21.15	6.97
Future	32.17	6.85	31.03	6.94	29.46	8.74	26.60	8.01	30.63	7.52
**DASS-21**										
Total score	14.79	11.16	18.83	12.42	22.24	13.63	27.75	14.54	19.31	13.03
Depression	4.97	4.43	6.14	4.76	7.46	5.36	9.30	5.51	6.39	5.01
Anxiety	4.29	4.05	4.95	4.40	6.14	4.70	7.94	5.40	5.27	4.57
Stress	5.53	4.37	7.74	4.91	8.65	5.10	10.51	5.37	7.65	5.05

*Descriptive statistics are reported for the untransformed variables*.

To identify the potential predictors of gambling behavior and gambling-related problems, gender, age and scores on FDIS, MCQ, CFC-14, and DASS-21 scales were input to a multiple regression analysis with SOGS-RA scores as the dependent measure. Results of hierarchical regression analysis (see Table 4) showed that, along with gender and age, dysfunctional impulsivity, anxiety, depression, short time horizon, and delay discounting significantly predicted gambling severity. The overall model explained about a third part of the total variance of the SOGS-RA [$R_{\text{adj}}^{2}$ = 0.273; *F*_(8, 1001)_ = 48.35; *p* < 0.001].

**Table 4:** **Summary of hierarchical regression analysis**.

**Variable**	**B**	***R^2^***	**Δ*R*^2^**	**β**	***t***	***p***	**VIF**
**STEP 1**							
Gender	−0.258	0.087	0.087	−0.270	−8.924	0.000	1.007
Age	0.034			0.145	4.792	0.000	1.007
**STEP 2**							
Gender	−0.267	0.193	0.106	−0.279	−9.827	0.000	1.008
Age	0.032			0.139	4.885	0.000	1.007
Dysfunctional Impulsivity	0.021			0.326	11.510	0.000	1.001
**STEP 3**							
Gender	−0.289	0.230	0.036	−0.302	−10.805	0.000	1.022
Age	0.030			0.129	4.637	0.000	1.010
Dysfunctional Impulsivity	0.018			0.281	9.880	0.000	1.057
DASS-21 Anxiety	0.021			0.198	6.893	0.000	1.076
**STEP 4**							
Gender	−0.289	0.250	0.020	−0.302	−10.920	0.000	1.022
Age	0.029			0.123	4.487	0.000	1.011
Dysfunctional Impulsivity	0.016			0.246	8.524	0.000	1.117
DASS-21 Anxiety	0.022			0.208	7.318	0.000	1.081
CFC-14 Future	−0.009			−0.146	−5.176	0.000	1.059
**STEP 5**							
Gender	−0.268	0.264	0.014	−0.281	−10.091	0.000	1.054
Age	0.030			0.127	4.663	0.000	1.012
Dysfunctional Impulsivity	0.013			00.199	6.492	0.000	1.278
DASS-21 Anxiety	0.020			0.195	6.880	0.000	1.093
CFC-14 Future	−0.010			−0.160	−5.701	0.000	1.074
CFC-14 Immediate	0.009			0.130	4.367	0.000	1.210
**STEP 6**							
Gender	−0.271	0.272	0.008	−0.283	−10.238	0.000	1.055
Age	0.028			0.120	4.414	0.000	1.018
Dysfunctional Impulsivity	0.012			0.186	6.052	0.000	1.299
DASS-21 Anxiety	0.012			0.115	3.146	0.000	1.853
CFC-14 Future	−0.010			−0.164	−5.873	0.000	1.076
CFC-14 Immediate	0.009			0.127	4.273	0.000	1.212
DASS-21 Depression	0.012			0.125	3.382	0.001	1.876
**STEP 7**							
Gender	−0.260	0.280	0.008	−0.272	−9.808	0.000	1.071
Age	0.025			0.105	3.840	0.000	1.047
Dysfunctional Impulsivity	0.012			0.186	6.100	0.000	1.299
DASS-21 Anxiety	0.011			0.108	2.939	0.003	1.862
CFC-14 Future	−0.010			−0.156	−5.600	0.000	1.084
CFC-14 Immediate	0.008			0.118	3.984	0.000	1.222
DASS-21 Depression	0.012			0.129	3.521	0.000	1.879
*k* Total score	0.037			0.090	3.236	0.001	1.066

*B, unstandardized coefficient; ΔR^2^, R square change; β, standardized regression coefficient; VIF, variance inflation factor*.

Finally, to analyze the developmental trajectories of gambling involvement, trait impulsivity, delay discounting, time perspective, and negative affective states, participants were divided into four age-groups (12–13, 14–15, 16–17, and 18–19 years, respectively). Subsequently, SOGS-RA scores across age-groups were analyzed by means of univariate ANOVA, whereas scores on the FDIS, MCQ, CFC-14, and DASS-21 subscales (within-participants variables) were submitted to repeated measures ANOVAs followed by Bonferroni *post-hoc* test, with gender, age group, and SOGS-RA classification as between-participants variables.

**Figure 1:**
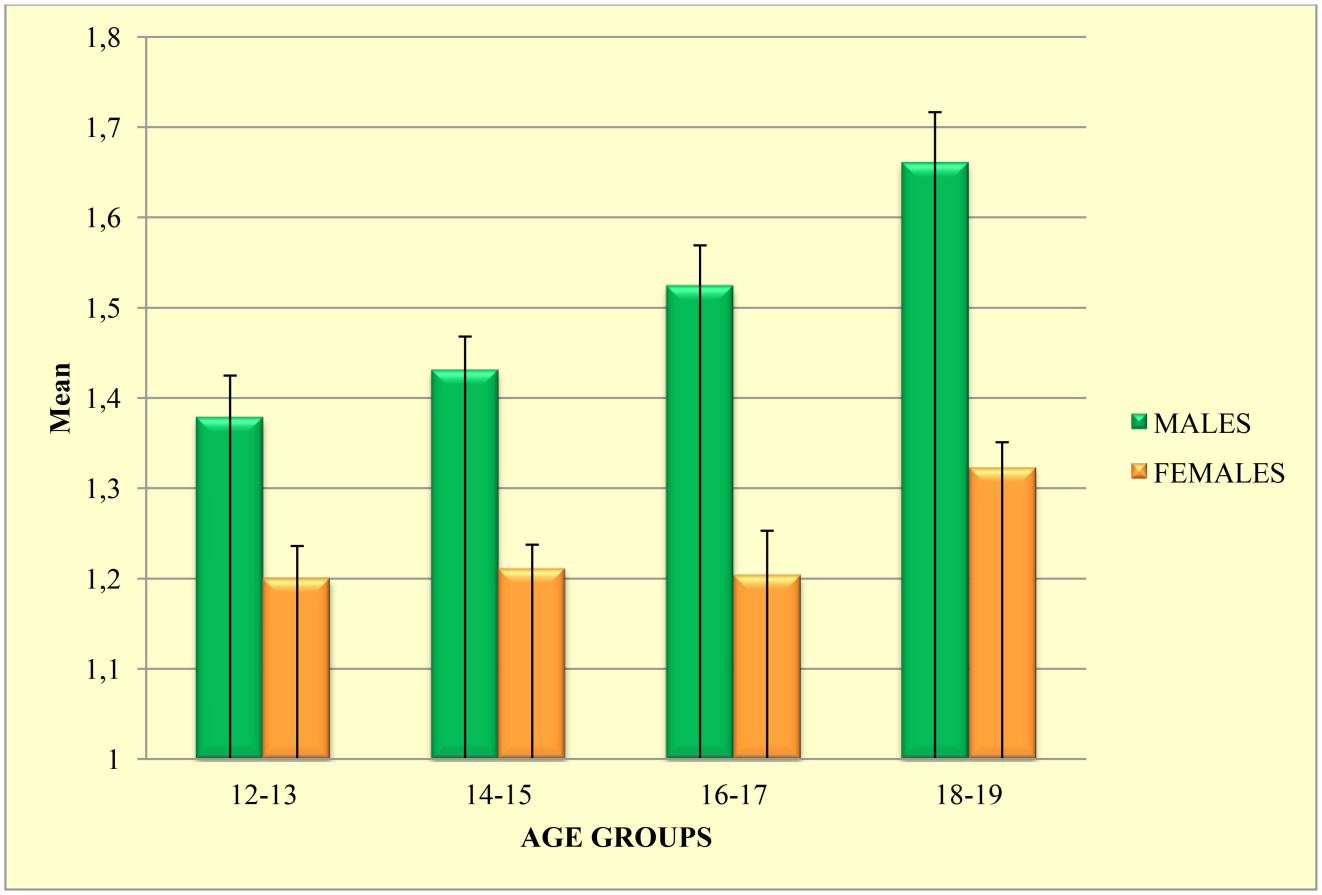
**SOGS-RA mean scores as a function of gender and age**. Error bars indicate standard error of the mean.

As far as SOGS-RA scores, a 2 (gender) × 4 (age group) ANOVA yielded significant main effects of gender [*F*_(1, 1002)_ = 77.66; *p* < 0.001; $\eta_{\text{p}}^{2}$ = 0.072] and age group [*F*_(3, 1002)_ = 8.24; *p* < 0.001; $\eta_{\text{p}}^{2}$ = 0.024], showing that gambling severity varies as a function of gender, with males reporting higher scores than females, and increases progressively with age (see Figure 1).

In regard to functional and dysfunctional impulsivity, mixed model ANOVA, with gender, age group, and SOGS-RA group entered as between-subjects factors, and FDIS subscales as within-subjects factor, yielded a main effect due to SOGS-RA classification [*F*_(3, 979)_ = 21.37; *p* < 0.001; $\eta_{\text{p}}^{2}$ = 0.061], indicating that both functional and dysfunctional impulsivity increased as a function of gambling severity. Furthermore, analysis yielded a significant interaction effect between gender and SOGS-RA classification [*F*_(3, 979_) = 2.97; *p* < 0.05; $\eta_{\text{p}}^{2}$ = 0.009], revealing that among non-gamblers females were less impulsive than males, whereas among problem gamblers females were more impulsive than males.

With regard to delay discounting, repeated measures ANOVA showed significant main effects of age group [*F*_(3, 979)_ = 4.30; *p* < 0.01; $\eta_{\text{p}}^{2}$ = 0.013] and SOGS-RA group [*F*_(3, 979)_ = 2.81; *p* < 0.05; $\eta_{\text{p}}^{2}$ = 0.009], indicating that scores increased with age and as a function of gambling severity.

With respect to time perspective, no significant between-subjects effect was observed. However, within-subjects contrasts revealed a significant interaction of CFC-14 scores and SOGS-RA classification [*F*_(1, 979)_ = 15.35; *p* < 0.001; $\eta_{\text{p}}^{2}$ = 0.045], reflecting the fact that Immediate scores increased, whereas Future scores decreased according to gambling severity.

As far as negative affectivity, mixed-model ANOVA identified significant main effects of gender [*F*_(1, 979)_ = 9.27; *p* < 0.01; $\eta_{\text{p}}^{2}$ = 0.009], with females obtaining higher DASS-21 scores than males, and SOGS-RA classification [*F*_(3, 979)_ = 18.44; *p* < 0.001; $\eta_{\text{p}}^{2}$ = 0.053], indicating that negative emotional states increased as a function of gambling severity.

Taken together, these results indicated that gambling severity contributes more than age in shaping the developmental trajectories of functional and dysfunctional impulsivity, delay discounting, time perspective, and negative affective states.

## Discussion

The present study is the first research that analyzes the interplay of self-reported functional and dysfunctional impulsivity, delay discounting, time perspective, and emotional negative states to gambling severity in adolescents. Previous research suggest the idea that problematic gambling, impulsivity, shortsightedness, and negative psychological states in adolescence are somewhat nested. However, and this is the novelty of our paper, to date no study had ever considered these constructs jointly.

On the whole, data from this study indicated that, relative to non-gamblers and non-problem gamblers, at-risk and problem gamblers showed higher levels of impulsivity, steeper delay discounting, shorter time horizon, and reported experiencing significantly higher levels of depression, anxiety, and stress.

In line with previous studies (van den Bos et al., 2013; Scholes-Balog et al., 2014; Welte et al., 2015; Raylu et al., 2016; for reviews see also Johansson et al., 2009; Griffiths, 2011; Donati et al., 2013), gender showed a significant negative relationship with SOGS-RA scores. However, no gender difference was observed with respect to the modes of gambling activities (offline vs. online). Interestingly, gambling routes did not vary as a function of age. Generally speaking, notwithstanding the advent of internet gambling, participants appeared to prefer traditional routes of gambling, probably because online gambling requires a credit card. However, especially striking is the amount of time and money spent on gambling activities. Just consider that a quarter of adolescent problem gamblers reported wasting between 10 and 50 Euros, and 14.5% of them more than 50 Euros in one day. In our opinion, future research should ask participants how they raise funds.

As far as impulsivity, results further support previous studies demonstrating that impulsivity, apart from the instruments used to asses it, remains one of the most robust feature associated with disordered gambling (MacKillop et al., 2014). What we first observed on adolescents dovetails with Maccallum et al. (2007), who found that, compared to normative data, adult pathological gamblers seeking treatment reported higher scores on both functional and dysfunctional impulsivity. More interestingly, our results showed that only dysfunctional impulsivity represents a significant predictor of severity of adolescent gambling involvement.

As regards to delay discounting, the results are in accordance with previous research demonstrating that pathological gamblers devalue or discount delayed rewards to a greater extent than non-gamblers and non-problem gamblers do (Petry and Casarella, 1999; e.g., Alessi and Petry, 2003; Madden et al., 2011; Michalczuk et al., 2011; Brevers et al., 2012; Miedl et al., 2012; Petry, 2012; Kräplin et al., 2014; see also Gray and MacKillop, 2014; Cosenza and Nigro, 2015; for a review see Wiehler and Peters, 2015; Cosenza et al., 2016; Nigro and Cosenza, 2016; Ciccarelli et al., 2016b).

In light of our results, adolescent gamblers show a similar shortsightedness by ignoring the future consequences of their actual behavior. More specifically, at-risk and problem gamblers appear to be more prone to focus on the immediate outcomes of their behavior than both non-gamblers and non-problem gamblers. This finding extends evidence obtained on both adult and adolescent samples (Hodgins and Engel, 2002; Whiteside et al., 2005; Toplak et al., 2007; Daugherty and Brase, 2010; MacLaren et al., 2012; Cosenza et al., 2014, 2016; MacKillop et al., 2014; Cosenza and Nigro, 2015; Ciccarelli et al., 2016a).

Since dysfunctional impulsivity was found to be strongly associated with the tendency to ignore hard facts when making decision (Dickman, 1990), it is no wonder that there are significant correlations among SOGS-RA, FDIS, and both CFC-14 and MCQ scores. It may be that high levels of dysfunctional impulsivity exacerbate the individual's inability to consider carefully the long-term future consequences of actions and to pay attention to one's own future, with all these impulsivity facets concurring to foster gambling addiction.

As with previous research (Lee et al., 2011; Hartmann and Blaszczynski, 2016; for reviews see Ciccarelli et al., 2017), the present study found that the more individuals have a problematic gambling involvement, the more they experience anxiety and depression. These results confirm the findings of previous studies demonstrating that among both adolescents and adults anxiety and depression co-occur with problematic gambling (Blaszczynski and McConaghy, 1989; Coman et al., 1997; Raylu and Oei, 2002; Kim et al., 2006; Barrault and Varescon, 2013; Martin et al., 2014; Estevez et al., 2015; Chinneck et al., 2016; Cunningham et al., 2016; Toneatto and Pillai, 2016; see also, Takamatsu et al., 2016). It may be that depression foregoes problem gambling, which serves to relieve negative emotions and to avoid problems (Blaszczynski and Nower, 2002) or that problematic gambling involvement increasingly leads to depressive symptoms due to the consequent social isolation and money problems (Dussault et al., 2011). Although it is difficult to determine whether anxiety and depression are primary, secondary, or concurrent with gambling, recently Raylu et al. (2016) have demonstrated that negative affectivity directly predicts gambling behavior.

Results of cross-sectional analyses indicated that gambling involvement increases as a function of gender and age. As depicted in Figure 1, the gambling involvement increases linearly with age among males, whereas among females the trend remains quite flat from 12 to 17 years, but picks significantly in late adolescence. This result corroborates the existence of a telescoping phenomenon, “whereby women as compared to men begin engagement in the behavior on average later in life than do men but the time between initial participation and development of a problem is shorter (or telescoped) in women as compared to men” (Potenza, 2013, p. S26).

As far as impulsivity, results indicated that the developmental trajectories of functional and dysfunctional impulsivity among adolescents are shaped mostly by the severity of gambling involvement. The same holds true for time perspective and delay discounting. Indeed, adolescent at-risk and problem gamblers appeared to devote less attention to the future, with more of the focus on the present (for similar results see Toplak et al., 2007; Cosenza and Nigro, 2015), and to have a weak orientation to the future also by choosing smaller but immediate rewards over larger but delayed rewards. Although some cross-sectional studies have demonstrated that in healthy adolescents delay discounting slightly declines in late adolescence (e.g., Green et al., 1994; Olson et al., 2007; Steinberg et al., 2009; see Albert and Steinberg, 2011 for a review), the results of cross-sectional analysis might suggest that gambling severity put the positive age-related changes across adolescence almost in the shade.

Finally, the results indicate that negative psychological states, namely anxiety and depression, increase as a function of gender and gambling involvement. These findings further support previous research reporting a stronger association between gambling severity and both depression and anxiety disorders in women than in men (Getty et al., 2000; Petry et al., 2005; Ste-Marie et al., 2006; Kessler et al., 2008; e.g., Desai and Potenza, 2008; Williams et al., 2012; see also Cunningham et al., 2016).

Given that anxiety and depression have been considered both precursors and consequences of problem gambling (see Hartmann and Blaszczynski, 2016), having found that female adolescents reported significantly greater levels of anxiety and depression suggests that gambling research, prevention, and treatment programs should consider carefully gender differences. In addition, since the combination of high impulsivity and emotional vulnerability contributes to foster the cycle of pathological gambling (e.g., McCormick et al., 1984), treatment protocols for gambling disorder should also take in account this underlying interplay. Indeed, as stressed by Blaszczynski and Nower (2002) and Hartmann and Blaszczynski (2016), the co-occurrence of emotional vulnerability and problematic gambling makes treatment more difficult. If this is true for adults, it is especially true for adolescents.

## Limitations

Although there are several strengths of the present study, including the large sample of participants, there are some limitations that should be considered when interpreting the present results. First, the current data are mainly based on self-report measures. In addition, it is to bear in mind that some authors questioned the validity of SOGS-RA (see Stinchfield, 2010 for a review), whereas other authors support the suitability of the instrument as a screening tool in adolescent populations (see Chiesi et al., 2013). Besides, it is worth to specify that the findings obtained are based on the general population of adolescents (12–19 years old), since no clinical group has been included in the study. Secondly, even if several studies demonstrated that there is no difference across hypothetical and potentially real rewards (e.g., Johnson and Bickel, 2002; Madden et al., 2003; Lagorio and Madden, 2005), delay discounting was evaluated using a behavioral measure that relies on hypothetical monetary choices. A final limitation is the use of cross-sectional sampling to analyze the developmental trajectories of gambling involvement, functional, and dysfunctional impulsivity, delay discounting, consideration of future consequences, and negative affectivity instead of a more appropriate longitudinal approach. Despite these limitations, to the authors' knowledge, the present study is the first to investigate the interplay of functional and dysfunctional impulsivity, time perspective, delay discounting, and negative affectivity on gambling severity among adolescents.

## Author contributions

Authors MC and GN together designed the study and wrote the protocol. Author MCi conducted literature searches and provided summaries of previous research studies. Author GN conducted the statistical analysis. Authors MC and GN wrote the first draft of the manuscript and all authors contributed to and have approved the final manuscript.

### Conflict of interest statement

The authors declare that the research was conducted in the absence of any commercial or financial relationships that could be construed as a potential conflict of interest.
